# Heightened Crescentic Glomerulonephritis in Immune Challenged 129sv Mice Is TGF-β/Smad3 Dependent

**DOI:** 10.3390/ijms22042059

**Published:** 2021-02-19

**Authors:** Yong Du, Chun Xie, Sneha Ravikumar, Jacob Orme, Li Li, Xin J Zhou, Chandra Mohan

**Affiliations:** 1 The Department of Biomedical Engineering, University of Houston, Houston, TX 77204-5060, USA; duyong717@gmail.com; 2 The Department of Internal Medicine, University of Texas Southwestern Medical Center, Dallas, TX 75390-0000, USA; chun.xie9@gmail.com (C.X.); sneharavi29@gmail.com (S.R.); jake.j.orme@gmail.com (J.O.); Li.Li@UTSouthwestern.edu (L.L.); JZhou@pbmlabs.com (X.J.Z.)

**Keywords:** anti-GBM nephritis, TGF-β/SMAD signaling, 129sv strain

## Abstract

The 129sv mouse strain is particularly sensitive to experimental immune-mediated nephritis. Previous studies have indicated that transforming growth factor-β (TGF-β) plays a critical role in both immune modulation and tissue fibrogenesis in various diseases and that its biological activities are exerted via the SMAD family. In this study, we aimed to determine whether TGF-β/SMAD signaling is essential for the development of immune-mediated nephritis in 129sv mice. Relative to C57BL/6J control mice with anti-glomeruli basement membrane (GBM) nephritis, 129sv mice with anti-GBM nephritis exhibited increased renal collagen deposition. Additionally, higher mRNA levels of pro-collagen and collagen IV, higher serum levels of active and total TGF-β1, and increased TGF-β1, TGF-βIIR, and phosphorylated SMAD expression were detected in these mice. Deletion of *Smad3* in 129sv mice ameliorated anti-GBM induced nephritis, including crescentic glomerulonephritis. Collectively, these findings indicate that the heightened experimental nephritis and fibrotic disease in the 129sv strain of mice are regulated by SMAD3, which could be a potential therapeutic target for immune-mediated nephritis.

## 1. Introduction

Transforming growth factor-β (TGF-β) is a potent, multi-functional cytokine with a broad spectrum of biological activities under both physiological and pathological conditions. The fibrogenic role of TGF-β in the pathogenesis of renal diseases, including lupus nephritis, has been well-established in various experimental models and human studies [1]. However, TGF-β blockade using a neutralizing antibody has been shown to worsen proteinuria in murine diabetic nephropathy and rat puromycin aminonucleoside nephropathy [2,3], implicating a complex role in renal disease progression. Indeed, TGF-β has anti-inflammatory and immunosuppressive functions in systemic immunity, but it is also a major inducer of local fibrosis in most types of kidney disease, including lupus nephritis [4]. Lower serum levels of TGF-β in active systemic lupus erythematosus (SLE) patients have been reported to be negatively correlated with disease severity and organ damage [5,6,7,8]. Importantly, TGF-β appears to be the most consistent intrinsic cytokine abnormality in SLE, independent of immunosuppressive drug usage [5]. Furthermore, elevated TGF-β expression has been detected in lupus patient renal tissue, and the renal cortical levels of *TGF-β1* mRNA have been reported to be correlated with histological activity [9]. Similar to these findings, it has been observed in NZB/W F1 lupus-prone mice that reduced TGF-β triggers immune dysregulation and auto-antibody production. Conversely, enhanced TGF-β expression leads to dysregulated tissue repair, progressive fibrogenesis, and eventual end-organ kidney damage [10]. Studies in both lupus patients and animal models indicate a dual role for TGF-β in disease pathogenesis and target organ damage [4,10].

Anti-glomeruli basement membrane (GBM) nephritis is characterized by acute renal dysfunction and the presence of serum anti-GBM auto-antibodies [11]. Compared to spontaneous lupus nephritis, anti-GBM nephritis exhibits a number of unique features, including its short time course and acute nephritis [4]. However, careful comparison of cytokines, chemokines, growth factors, and adhesion molecules involved in these two diseases highlights shared pathogenic pathways [4,12]. Similar to observations in murine lupus models, TGF-β blockade also ameliorates renal dysfunction and histological progression in anti-GBM nephritis [13]. Likewise, TGF-β receptor II (TGF-β-RII) deficiency prevents anti-GBM nephritis [14].

One interesting aspect of the experimental anti-GBM nephritis mouse model is that different strains are inherently more sensitive. Among more than 20 strains studied, we found that the 129sv mouse strain is more susceptible to developing nephritis, whereas the C57BL/6J strain is relatively resistant [15,16]. The reason for this enhanced susceptibility remains unknown, and the potential role of TGF-β has not been examined.

TGF-β exerts its biological functions through its downstream signaling molecules, notably the SMAD family of proteins [17]. After TGF-β binds to TGF-β-RII, TGF-β receptor I (TGF-β-RI) is recruited to form a heterotetrameric complex. Activated TGF-β-RI constitutively interacts with and phosphorylates downstream SMAD family members that transduce the signal to the nucleus [17,18]. Thus, overexpression of SMAD7, a negative regulator of TGF-β/SMAD signaling, confers protection from anti-GBM nephritis [19]. It is now clear that SMAD3 is critical for the pro-fibrotic activity of TGF-β [1,17]. Whether the contribution of TGF-β to the pathogenesis of nephritis is SMAD3 dependent and whether the enhanced anti-GBM nephritis in 129sv mice is driven by SMAD3 are currently unknown. Therefore, we designed this study to address these questions. The gene and protein expression levels of TGF-β were compared between B6 and 129sv mice with anti-GBM nephritis. Mice with *Smad3* gene deficiency were used to determine whether the enhanced anti-GBM nephritis in 129sv mice is SMAD3 dependent. We found higher serum levels of active and total TGF-β1, and increased TGF-β1, TGF-βIIR, and phosphorylated SMAD3 expression in 129sv anti-GBM nephritic mice. Moreover, mice deficient in *Smad3* were protected from anti-GBM disease, indicating that the more severe nephritis and fibrotic disease observed in the 129sv strain of mice were regulated by SMAD3.

## 2. Results

### 2.1. 129sv Mice Are More Susceptible to Anti-GBM Nephritis Than C57BL/6J Mice

**Figure 1:**
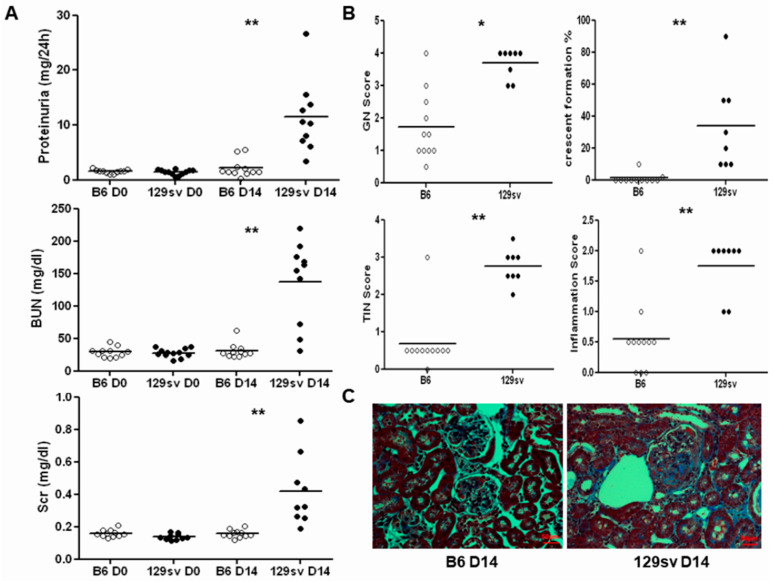
Clinical and pathological features of 129sv and C57BL/6J mice after anti-glomeruli basement membrane (GBM) serum challenge. Renal function and pathological changes in 129sv and C57BL/6J mice after anti-GBM serum challenge. (**A**) 24 h proteinuria (PU), blood urea nitrogen (BUN), and serum creatinine (Scr) levels in 129sv and C57BL/6J mice at baseline and D14. (**B**) Renal pathological changes, including the glomerulonephritis (GN) score, percentage of crescent formation, tubule-interstitial (TIN) score, and inflammation score in 129sv and C57BL/6J mice (n = 8–10 mice per group, * *p* < 0.05, ** *p* < 0.001, two-tailed *t*-test). (**C**) Masson’s trichrome staining showing increased collagen deposition in 129sv mice with anti-GBM nephritis (200×). The scale-bar shown = 50 µm.

Similar to our previous study [15], we found that 129sv mice exhibited more severe renal disease than C57BL/6J mice after anti-GBM serum challenge (Figure 1). Both groups exhibited similar baseline proteinuria, blood urea nitrogen (BUN), and serum creatinine levels (Figure 1A). On D14 after anti-GBM serum challenge, 129sv mice exhibited higher proteinuria and BUN levels (11.45 ± 6.53 vs. 2.14 ± 1.68 g/24 h, 136.75 ± 63.73 vs. 30.98 ± 11.44 mg/dL, *p* < 0.001, respectively) as well as significantly elevated serum creatinine levels compared with anti-GBM nephritis C57BL/6J controls (0.42 ± 0.12 vs. 0.16 ± 0.02 mg/dL, *p* = 0.001). Similarly, 129sv mice exhibited severe histological changes relative to the C57BL/6J controls. An average glomerulonephritis (GN) score of 3.69 ± 0.16 was observed in 129sv anti-GBM nephritis mice, compared with 1.72 ± 0.32 in the C57BL/6J controls (*p* < 0.0001). Almost 30% of the glomeruli of the 129sv mice exhibited crescent formation (33.75 ± 9.99 glomeruli per section). In contrast, C57BL/6J mice were relatively resistant to crescent formation (1.09 ± 096 glomeruli per section, *p* = 0.0013). The 129sv mice also displayed severe tubule-interstitial changes and inflammatory cell infiltration (Figure 1B). Most importantly, Masson’s trichrome staining showed more collagen deposition in 129sv mice with anti-GBM nephritis than the C57BL/6J challenged controls (Figure 1C).

### 2.2. TGF-β1 and Col1a1/Col4a1 mRNA Expression Are Elevated in the Kidneys of 129sv Mice

**Figure 2:**
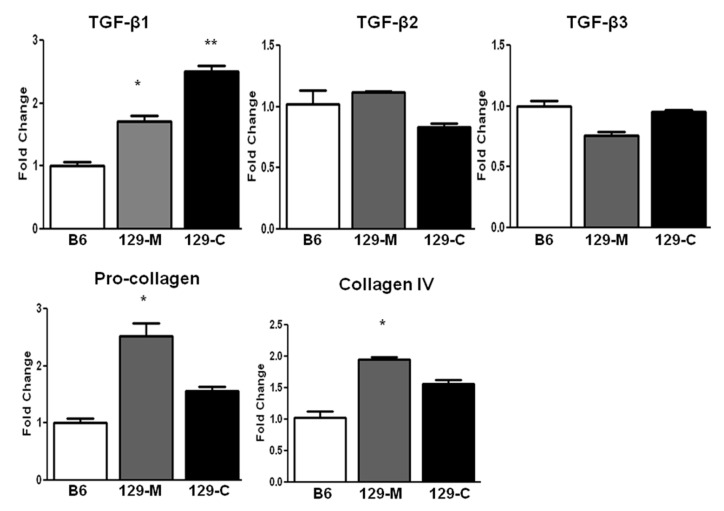
Renal *Tgf-β* and collagen mRNA levels in the kidneys of 129sv and C57BL/6J mice with anti-GBM nephritis. Real-time PCR analysis of renal cortical and medullary *Tgf-β1*, *Tgf-β2*, and *Tgf-β3* mRNA expression in 129sv and C57BL/6J mice at D21 after anti-GBM challenge. 129-M represents the 129sv mice renal medulla, while 129-C represents renal cortex. Only *Tgf-β1* expression was higher in the cortex and medulla of 129sv mice with anti-GBM nephritis (expression of C57BL/6J mice normalized to 1.0). Increased pro-collagen (*Col1a1*) and collagen IV (*Col4a1*) mRNA levels were also detected in the renal tissues of 129sv mice with anti-GBM nephritis compared with those of the control mice (n = 5 mice per group, * *p* < 0.05, ** *p* < 0.001, two-tailed *t*-test).

The transcription levels of TGF-β family member genes, *Tgf-β1*, *Tgf-β2*, and *Tgf-β3*, were compared between groups using real-time PCR. Only the expression of *Tgf-β1* was significantly elevated in the kidneys of the 129sv mice compared with the C57BL/6J mice (renal cortex, 2.47-fold increase; medulla, 1.84-fold increase; *p* = 0.01, normalized to 1). However, *Col1a1* and *Col4a1* were also increased in the medullas of the 129sv mice compared with the control mice (Figure 2).

### 2.3. TGF-β1 Protein Levels Are Elevated in Both the Sera and Renal Tissue of 129sv Mice with Anti-GBM Nephritis

**Figure 3:**
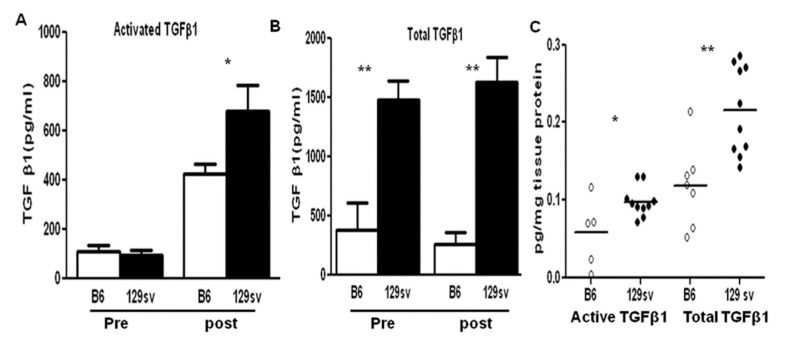
Sera and renal active and total TGF-β1 levels in 129sv and C57BL/6J mice with anti-GBM nephritis. (**A**) Active TGF-β1 and (**B**) total TGF-β1 serum levels. (**C**) Active and total TGF-β1 levels in renal tissues. 129sv mice exhibited higher total TGF-β levels at baseline and D21 compared to the C57BL/6J control mice. Total and active TGF-β1 were measured in renal tissues and eluates by ELISA. As in sera, diseased renal tissues from 129sv mice exhibited significantly higher levels of both total and active TGF-β1 (n = 8–10 mice per group, * *p* < 0.05, ** *p* < 0.001, two-tailed *t*-test).

The serum levels of total and active TGF-β1 between the 129sv and C57BL/6J mice were compared, prior to and after anti-GBM serum challenge (n = 10–12 mice per group). Compared with the control mice, 129sv mice exhibited higher baseline total TGF-β1 serum levels (*p* < 0.0001, Figure 3B). Additionally, these mice exhibited significant increase after anti-GBM serum challenge. Both groups exhibited similar baseline levels of active TGF-β1, but 129sv mice exhibited increased expression of active TGF-β1 on D14 after anti-GBM serum challenge. Furthermore, the levels of both total and active TGF-β1 were also increased in the renal eluates from 129sv mice with anti-GBM nephritis, but not in the renal eluates of the C57BL/6J mice (n = 7–10, Figure 3C).

### 2.4. TGF-β1/SMAD Signaling Molecules Are Increased in the Kidneys of 129sv Mice

**Figure 4:**
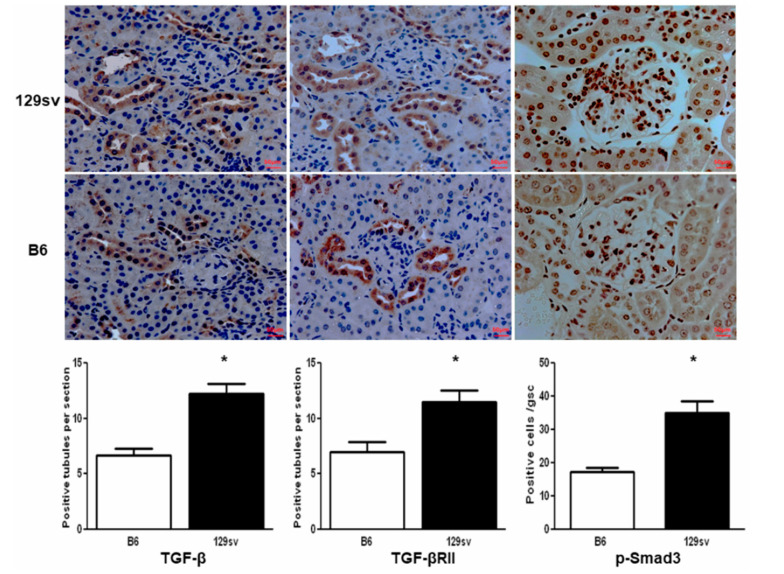
TGF-β1, TGF-β1-RII, and p-Smad3 expression in the kidneys of 129sv and C57BL/6J mice with anti-GBM nephritis. Immunohistochemical staining of TGF-β1, TGF-β1-RII, and p-Smad3 at D21 (top: 129sv kidney, bottom: C57BL/6J kidney, 400×). Quantification (lower panel). Data are presented as the mean ± standard error of the mean (SEM) (n = 5 mice per group, * *p* < 0.05, two-tailed *t*-test). Both TGF-β1 and TGF-β1-RII are mainly expressed in renal tubular epithelial cells, whereas p-Smad3 expression can be seen in both the glomeruli and tubules. The scale-bar shown = 50 μm.

Immunohistochemical analysis revealed the increased expression of TGF-β1 and its downstream pathway molecules, TGF-β1-RII and p-Smad3, in the diseased kidneys of the 129sv mice (*p* < 0.05 vs. the control group, n = 5 mice per group, Figure 4). TGF-β1 and TGF-β1-RII were mainly expressed in and around the tubular epithelial cells, whereas p-Smad3 was observed within the nuclei of both tubular and glomerular cells.

### 2.5. SMAD3 Deficiency Protects 129sv Mice from Anti-GBM Nephritis

**Figure 5:**
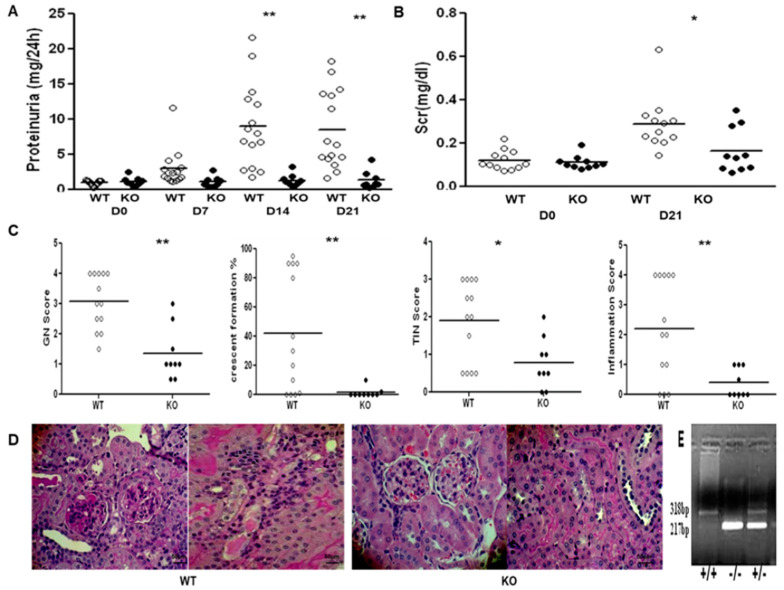
SMAD3 deficiency reduces disease in 129sv mice after anti-GBM serum challenge. (**A**) 24 h proteinuria (PU) changes between 129.*Smad3*^−/−^ and wild-type (WT) 129sv mice at different time points. After anti-GBM serum challenge, WT mice exhibited significantly increased 24-h PU, whereas 129.*Smad3*^−/−^ mice exhibited no changes relative to baseline (n = 9–13, ** *p* < 0.001 at D14 and D21, two-tailed *t*-test). (**B**) Serum creatinine levels between 129.*Smad3*^−/−^ and WT mice at baseline and D21 after anti-GBM serum challenge. Compared with WT mice, 129.*Smad3*^−/−^ mice exhibited lower serum creatinine (Scr) levels at D21 (n = 9–13, * *p* < 0.05, two-tailed *t*-test). Consistent with reduced renal dysfunction, SMAD3 deficiency also improved renal pathology after anti-GBM serum challenge, as shown in (**C**) for the 129.*Smad3*^−/−^ and WT littermate control mice after anti-GBM serum challenge (left to right: glomerulonephritis (GN) score, percentage of crescent formation, tubulointerstitial (TIN) score, and inflammation score; **, *p* < 0.001, two-tailed *t*-test). Representative glomerular (left) and tubulointerstitial (right) changes in WT and 129.*Smad3*^−/−^ mice (200×) are shown in (**D**). Shown data represent findings from 9–13 mice per group. Representative genotyping image from the experimental and control strains is shown in (**E**), where the 318 bp band represents the WT mouse and the 217 bp band represents the Smad3 KO mouse. The scale-bar shown = 50 μm.

Because the above findings showed enhanced expression of TGF-β1 and its signaling molecules in the kidneys of 129sv mice with anti-GBM nephritis, we hypothesized that blockade of TGF-β/SMAD signaling may protect 129sv mice from anti-GBM nephritis. Renal function and pathological changes were compared between 129.*Smad3*^−/−^ and wild-type (WT) control mice. No significant changes were observed in proteinuria (1.08 ± 0.64 vs. 1.28 ± 1.27 g/24 h) or serum creatinine levels (0.109 ± 0.03 vs. 0.164 ± 0.104 mg/dL, on D0 and D21, respectively) in the 129.*Smad3*^−/−^ mice 24 h after anti-GBM serum challenge; however, significant changes in proteinuria (D0, 0.90 ± 0.36; D14, 8.99 ± 5.95; D21, 8.42 ± 5.54 g/24 h, *p* < 0.0001) and increased serum creatinine levels (D0, 0.121 ± 0.04; D14, 0.288 ± 0.123 mg/dL, *p* = 0.018) were observed in the WT control mice, indicating severe renal disease. Furthermore, 129.*Smad3*^−/−^ mice exhibited a significantly lower GN score (1.33 ± 0.87) as well as dramatically reduced crescent formation (0.22 ± 0.44) than that of WT mice (2.89 ± 0.96; 1.44 ± 1.33, respectively, *p* < 0.05, Figure 5C). The TI score of 129.*Smad3*^−/−^ mice was 0.74 ± 0.46, significantly lower than that of WT mice (1.89± 1.16, *p* < 0.05, Figure 5C). Reduced inflammatory cell infiltration was also observed in 129.*Smad3*^−/−^ mice, compared with WT (0.39 ± 0.46 vs. 2.14 ± 1.63, *p* < 0.05, Figure 5C). Collectively, these data suggest that crescentic glomerulonephritis induced by anti-GBM serum in 129sv mice is SMAD3-dependent, underscoring the critical role of SMAD3 in the development of nephritis.

## 3. Discussion

TGF-β plays a critical role in various renal diseases, both as a pro-fibrotic cytokine and multi-functional immunomodulator. The present study revealed increased levels of TGF-β1 and its downstream signaling molecules in 129sv mice with experimentally induced anti-GBM nephritis, suggesting a role in the heightened susceptibility of these mice to immune complex-induced glomerulonephritis. Furthermore, deficiency of SMAD3, a key effector of TGF-β family signaling, protected these mice from immune complex-induced renal damage.

The pro-fibrotic role of TGF-β in renal diseases has been widely recognized; however, it has also been demonstrated as a pleiotropic cytokine with significant anti-inflammatory and immunosuppressive properties. A wasting syndrome associated with multifocal inflammation has been demonstrated in TGF-β1-knockout mice [20]. Systemic TGF-β overexpression has been shown to have an inhibitory effect in various autoimmune diseases, including spontaneous lupus nephritis [21], autoimmune encephalomyelitis [22], insulitis in NOD mice [23], and erosive arthritis [24]. These examples support a dual function of TGF-β in both SLE patients and spontaneous lupus nephritis models, as a reduction in circulating TGF-β allows immune intolerance, whereas enhanced local TGF-β triggers end-organ fibrogenesis [4,10].

Compared to the relatively clear role of TGF-β in lupus, its role in anti-GBM nephritis remains unclear. Early studies revealed that neutralizing TGF-β with antibodies or through TGF-β blockade in T cells ameliorated renal dysfunction and histological progression in anti-GBM nephritis [13,25]. Similarly, mice with TGF-β-RII deficiency exhibit protection against renal injury in crescentic glomerulonephritis [14]. However, it has been reported that mice with latent TGF-β overexpression in the skin display protection against anti-GBM crescentic glomerulonephritis and renal fibrosis in obstructive kidney disease [26,27]. The same group has also recently reported the important but diverse role of TGF-β-RII in regulating renal fibrosis and inflammation. They found that disruption of TGF-β-RII inhibited severe tubulointerstitial fibrosis in the unilateral ureteral obstructive (UUO) nephropathy model through the TGF-β/SMAD3 signaling pathway, whereas deletion of TGF-β-RII enhanced renal inflammation in a TGF-β/SMAD3-independent manner [28], alluding to the complex role of TGF-β in renal diseases.

Strain variation is a feature of the anti-GBM nephritis model, with the 129sv strain exhibiting enhanced susceptibility to immune-mediated nephritis [15,16]. Given the reported role of TGF-β in a variety of chronic renal diseases, including lupus nephritis and anti-GBM nephritis, we aimed to determine whether TGF-β and its downstream SMAD signaling molecules were essential for the renal damage observed in 129sv mice with anti-GBM nephritis. Indeed, the renal expression of TGF-β1 was higher in 129sv mice after anti-GBM serum challenge (Figure 3C and Figure 4). Higher total and active TGF-β serum levels were also found in 129sv mice with anti-GBM nephritis compared with the more resistant C57BL/6J strain (Figure 3A,B). These findings suggest that increased TGF-β signaling in 129sv mice may contribute to their enhanced susceptibility to anti-GBM nephritis. 

TGF-β1 signals via a complex of two membrane-bound receptor serine/threonine kinases that recruit and phosphorylate SMAD2 and SMAD3. Once phosphorylated, SMAD2 and SMAD3 oligomerize with SMAD4 and translocate into the nucleus to regulate transcriptional events [1,17]. Using SMAD3-deficienct mice, we demonstrate SMAD3 is essential for the development of immune-mediated nephritis in 129sv mice, as we observed reduced proteinuria and serum creatinine levels, as well as a lesser extent of renal pathology, in 129.*Smad3*^−/−^ mice compared with WT controls (Figure 5). Our findings, as well as those of previous reports, support a critical role for TGF-β/SMAD signaling in the pathogenesis of renal disease and that targeting SMAD3 appears to be an attractive therapeutic option for immune-mediated nephritis, including lupus nephritis and anti-GBM nephritis.

Several strategies have been developed to combat TGF-β/SMAD signaling-induced fibrosis, including small molecule inhibitors for the receptor serine/threonine kinase [29,30], neutralizing antibodies [31], decorin [32,33], SMAD7 overexpression [34], etc. These approaches have been demonstrated to inhibit renal fibrosis and inflammation in various rodent models. Similar to these findings, our results show that SMAD3 deficiency can protect against antibody-induced nephritis in mice, adding to the growing attractiveness of this signaling axis as a therapeutic target in nephritis. However, caution needs to be exercised since this axis may have opposing roles in systemic immunity versus end-organ disease, as discussed elsewhere [4,10]. In this context, the systemic versus end-organ activity of the TGF-β/SMAD3 axis needs to be carefully evaluated in the specific human disease we are targeting before planning treatment trials. Finally, renal targeting of nanomedicine bearing inhibitors of this axis, in order to confine treatment to the kidneys, also warrants exploration.

## 4. Materials and Methods

### 4.1. Animals and Anti-GBM Nephritis 

C57BL/6J and 129sv mice were obtained from the Jackson Laboratory (Bar Harbor, ME, USA). The 129.Smad3 knockout strain was a kind gift from Dr. Jonathan Graff, UT Southwestern Medical Center, Dallas, TX [35]. All mice were housed in a pathogen-free environment and were handled according to institutional IACUC guidelines. The animal use protocol was approved by the Institutional Animal Care and Use Committee at the University of Houston (MOHAN-APN04-17). Female mice (6–8 weeks old) were used for the study. A standard protocol was used for inducing anti-GBM nephritis, as previously described [15,16]. Briefly, mice were immunized on day 0 (D0) with rabbit IgG (250 μg/20 g of mouse body weight, Sigma #I5006) and complete Freund’s adjuvant (CFA, 100 µL/20 g of body weight, Sigma #F5881). At D5, these mice were administered a single dose of rabbit anti-mouse GBM serum (160 μL/20 g mouse body weight) via intravenous injection. For the next 24 h, mice were placed in metabolic cages with free access to water for urine sample collection. Urinary protein concentration was determined using the Coomassie Plus Protein Assay Kit (Pierce, Rockford, IL, USA). Blood was collected and serum creatinine (Scr) was measured using the P/ACE MDQ Capillary Electrophoresis System with a photodiode array detector (Beckman-Coulter, Fullerton, CA, USA). Serum BUN level was determined using urea nitrogen detecting reagents from Sigma (St. Louis, MO, USA). All animals were sacrificed on D21, and the kidneys were processed for pathological evaluation, renal eluates, and RNA isolation (cortical and medullary).

### 4.2. Reagents

Anti-GBM serum was generated by LAMPIRE Biological Laboratories (Everett, PA, USA) [15]. Rabbit anti-mouse TGF-β monoclonal antibody (mAb), anti-TGF-β-RII mAb, and anti-phosphorylated SMAD3 (p-Smad3) mAb were purchased from Abcam (Cambridge, MA, USA). TGF-β ELISA kit was obtained from R&D Systems (Minneapolis, MN, USA). A universal rabbit/mouse HRP kit was obtained from DAKO Company (Glostrup, Denmark). 

### 4.3. Genotyping of 129.smad3^−/−^ Mice and Renal Tgf-β and Collagen mRNA Level Detection Using Real-Time PCR

Genomic DNA was isolated from the tails of 129.*Smad3*^−/−^ mice using protease K and was subjected to PCR. The following primer sequences were used for genotyping: P1: 5′-TGG ACT TAG GAG ACG GCA GTC C-3′; P2: 5′-CTT CTG AGA CCC TCC TGA GTA GG-3′; P3: 5′-CTC TAG AGC GGC CTA CGT TTG G-3′. The reaction mixture contained 1× PCR buffer, 1.5 mmol/L MgCl_2_, 200 mmol/L deoxynucleotide triphosphates (dNTPs), 1 unit TaqDNA polymerase (MBI), 10 pmol of each primer, and 100–200 ng genomic DNA. The PCR amplification reaction consisted of a cycle at 94 °C for 10 min followed by 35 cycles of denaturation at 94 °C for 30 s, annealing at 55 °C for 30 s, and extension at 72 °C for 45 s. A final extension was performed at 72 °C for 5 min. Products were then electrophoresed on a 2% agarose gel containing ethidium bromide (1.5 μg/mL). 

Total RNA for RT-PCR analysis was isolated from the renal cortex or medulla tissues using TRIZOL (Qiagen, Valencia, CA, USA) and quantified using a spectrophotometer (Thermo-Fisher Scientific Inc., Wilmington, DE, USA). Real-time PCR primer sequences for *Tgf-β1*, *Tgf-β2*, *Tgf-β3*, collagen IV (*Col4a1*), and pro-collagen (*Col1a1*) were previously reported [10]. All real-time PCR reactions were performed using Fast SYBR Green Master Mix (Applied Biosystems) on a Bio-Rad CFX96 Real-Time System (Bio-Rad, Hercules, CA, USA). Gene expression was normalized to *Gapdh* expression using the standard ΔCT method.

### 4.4. Pathology and Immunohistochemistry

Formalin-fixed, paraffin-embedded kidney sections were stained with hematoxylin and eosin (H&E) and periodic acid Schiff (PAS). Sections were examined for pathological changes in the glomeruli, tubules, or interstitial areas in a blinded fashion. For glomeruli, hypertrophy, proliferative changes, crescent formation, hyaline deposits, fibrosis/sclerosis, and basement membrane thickening were graded on a scale of 0–4 [15,16]. At least 100 glomeruli per section were used to determine the percentage of glomeruli with crescent formation. Likewise, tubulo-interstitial injury was graded on a scale of 0–4 based on tubular atrophy, inflammatory infiltrates, interstitial fibrosis, and the severity of tubulo-interstitial nephritis (TIN) [15,16]. The inflammatory score was also evaluated by a renal pathologist in a blinded manner using a well-established histology scoring system based on H&E and PAS stained sections, as detailed in our earlier manuscripts [15,16,36,37,38].

Renal expression of TGF-β, TGF-βR-II, and p-Smad3 was detected by immunohistochemistry. Briefly, formalin-fixed, paraffin-embedded kidney tissues were cut into 4 µm thick sections. After deparaffinization and rehydration, sections were boiled in 0.1 M citrate buffer for antigen retrieval. The sections were incubated with primary antibodies at the appropriate dilutions overnight at 4 °C. The next day, the slides were washed with phosphate-buffered saline (PBS), incubated with rabbit anti-mouse/rabbit secondary antibody for 2 h, and finally stained with 3′,3′-diaminobenzidine (DAB; Abcam). For TGF-β and TGF-β-RII staining, sections were counter-stained with hematoxylin to visualize cell nuclei. A semi-quantitative method was used to evaluate the expression of TGF-β and TGF-β-RII. Briefly, the number of positive-stained tubules was counted in at least 10 random fields per section. For p-SMAD, the positive cell number was counted in at least 30 glomeruli per section.

### 4.5. ELISA

Serum and renal eluate TGF-β expression was determined using a commercial mouse TGF-β ELISA kit from R&D according to the manufacturer’s instructions. Briefly, protein samples were acidified with 1 N HCl and neutralized with 1.2 N NaOH/0.5 M HEPES to determine the amount of total TGF-β, whereas the levels of active TGF-β1 were analyzed in unacidified samples. Tissue protein levels of TGF-β1 were normalized to total protein concentrations assayed using a bicinchoninic acid (BCA) protein assay reagent (Abcam, #ab253410, Cambridge, MA, USA).

### 4.6. Statistical Analysis

All data were expressed as mean ± SD. Comparison between two groups was performed using a parametric Student’s *t* test or non-parametric Mann–Whitney U test where indicated. A 2-tailed *p* value < 0.05 was considered statistically significant. All statistical analyses were performed using GraphPad Prism (V.10.0, GraphPad, CA, USA).

## Data Availability

No new data were created or analyzed in this study. Data sharing is not applicable to this article.
